# Case report: Mild leukoencephalopathy caused by a new mutation of *NOTCH3* gene

**DOI:** 10.1097/MD.0000000000033289

**Published:** 2023-03-24

**Authors:** Yuxiang Qi, Hairong Li, Ling Yu

**Affiliations:** a Department of Neurology, Shengli Oilfield Central Hospital, Dongying, Shandong, China.

**Keywords:** CADASIL, gene mutation, leukoencephalopathy, *NOTCH3*

## Abstract

Cerebral autosomal dominant arteriosis with subcortical infarction and leukoencephalopathy (CADASIL) is a single-gene small-vessel disease of the brain characterized by migraine, recurrent ischemic stroke, psychiatric disorders, progressive cognitive decline, and occasional intracerebral hemorrhage.^[1]^ *NOTCH3* was identified as a pathogenic gene for CADASIL.^[2]^ The *NOTCH3* gene encodes a membrane-bound receptor protein, and to date, several different *NOTCH3* gene mutations have been identified.^[3]^ Here, we report a case of CADASIL with a heterozygous mutation c.931T > G (thymine > guanine) on the exon region of the *NOTCH3* gene, resulting in an amino acid change p.C311G (cysteine > glycine).

**Case report:**

We report a case of a female patient with CADASIL whose genetic sequencing revealed a mutation in the *NOTCH3* gene. However, this patient did not exhibit any of the typical clinical findings of CADASIL but the patient’s cerebral magnetic resonance imaging was consistent with the characteristic findings of CADASIL.

**Conclusions:**

This case reminds us that mutations caused by different mutation sites present different clinical symptoms.

## 1. Introduction

*NOTCH3* gene mutation is associated with cerebral autosomal dominant arteriosis with subcortical infarction and leukoencephalopathy (CADASIL).^[4]^ *NOTCH3* gene is mainly expressed in smooth muscle cells of arterial vessels.^[5]^ *NOTCH3* protein belongs to *NOTCH3* receptor family, which extracellular region contains 34 epidermal growth factor like (EGF-like) repeats. Each EGF-like repeat is approximately 40 amino acids in length and contains 6 cysteine residues, which are pairwise bound by 3 disulfide bonds and play an important role in maintaining protein stability.^[6]^ CADASIL is the most familiar genetic disease caused by *NOTCH3* gene mutation. CADASIL mainly affects young and middle-aged people, with recurrent subcortical ischemic stroke, migraine, cognitive and affective disorders, and epilepsy as the main clinical manifestations.^[7]^ CADASIL can be disagnosed by the presence of mutations on 23 exons of *NOTCH3* gene or deposition of GOM or NOTCH protein in arteriolar smooth muscle.^[8]^

## 2. Case report

A 47-year-old female patient was treated in the Dermatology Department of Shengli Oilfield Central Hospital in December 2020 due to neck herpes zoster. The patient is a professional and technical worker with a junior college education level. She used to be healthy and she is an only child in her family. Diagnosis on admission showed that there were several clusters of miliary to soy-sized cuticular herpes in the skin of the patient’s right lateral neck but there is no herpes in the right external auditory canal. Herpes content is clear, without pus and blood, and the boundary is relatively clear. The rash was banded along the unilateral nerve distribution area.

**Figure 1.**
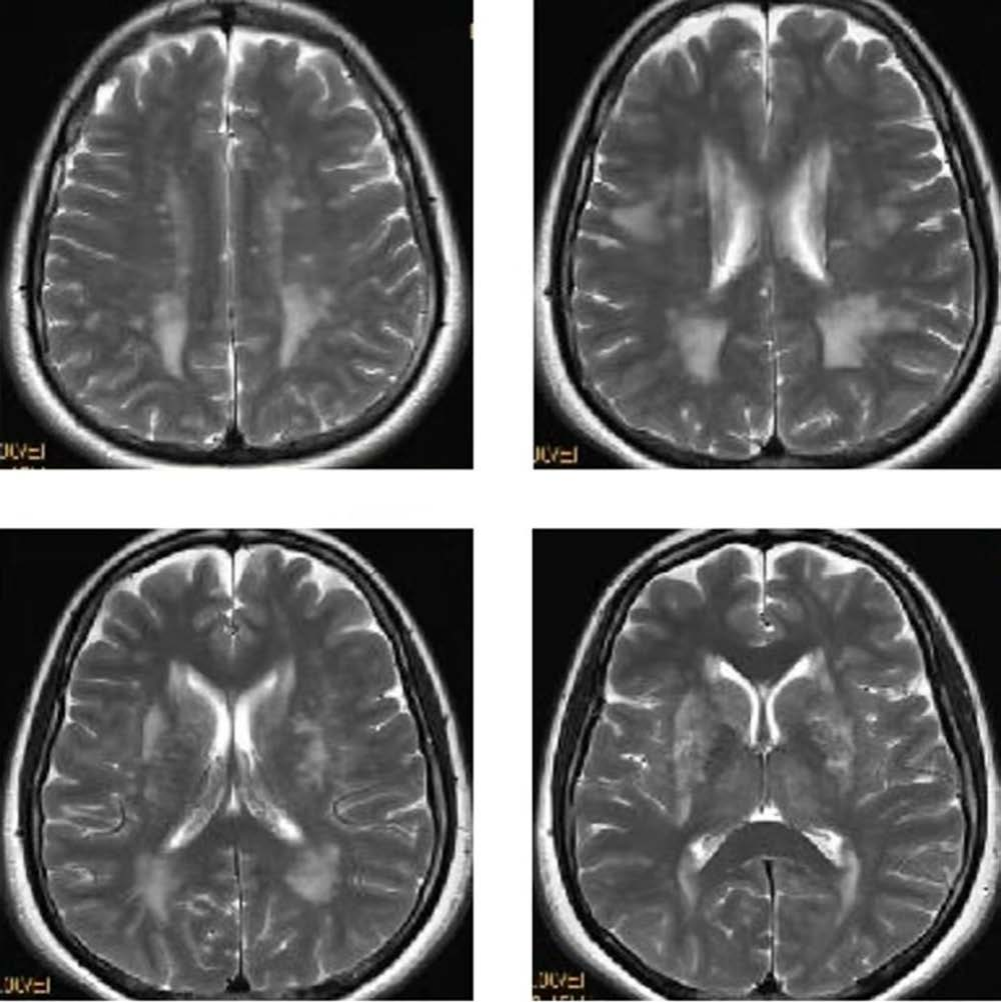
Cerebral MRI images of the patient’s brain. MRI = magnetic resonance imaging.

During hospitalization, the patient presented with right occipital pain. In order to clarify the cause, intracranial organic lesions were excluded, and improving cerebral magnetic resonance imaging (MRI) examination. MRI examination results are shown in Figure 1. Carefully inquire about her medical history, the results are as follows: The patient has a history of neurodermatitis for several years, and the condition is stable; The patient has no history of migraine, acute cerebrovascular disease and epilepsy; The patient has no history of CO poisoning and exposure to toxic substances such as heavy metals, benzene and organophosphorus pesticides; Both parents of the patient are healthy without a family history of hereditary diseases. In addition, the patient’s neurological examination showed no obvious positive signs. Improving the mini-mental state examination score = 29 and improving the Montreal cognitive assessment score = 28. Improving inspection of laboratory indexes, including antinuclear antibody spectrum, antivasculitis antibody spectrum, 4 items before operation (AIDS, syphilis, hepatitis B virus, hepatitis C virus), thyroid function and thyroid antibody, serum vitamin B12 level, folic acid level, serum immunoglobulin, complement, liver and kidney function, blood glucose, blood lipids and so on. No significant abnormalities were observed in the above indexes.

**Figure 2.**
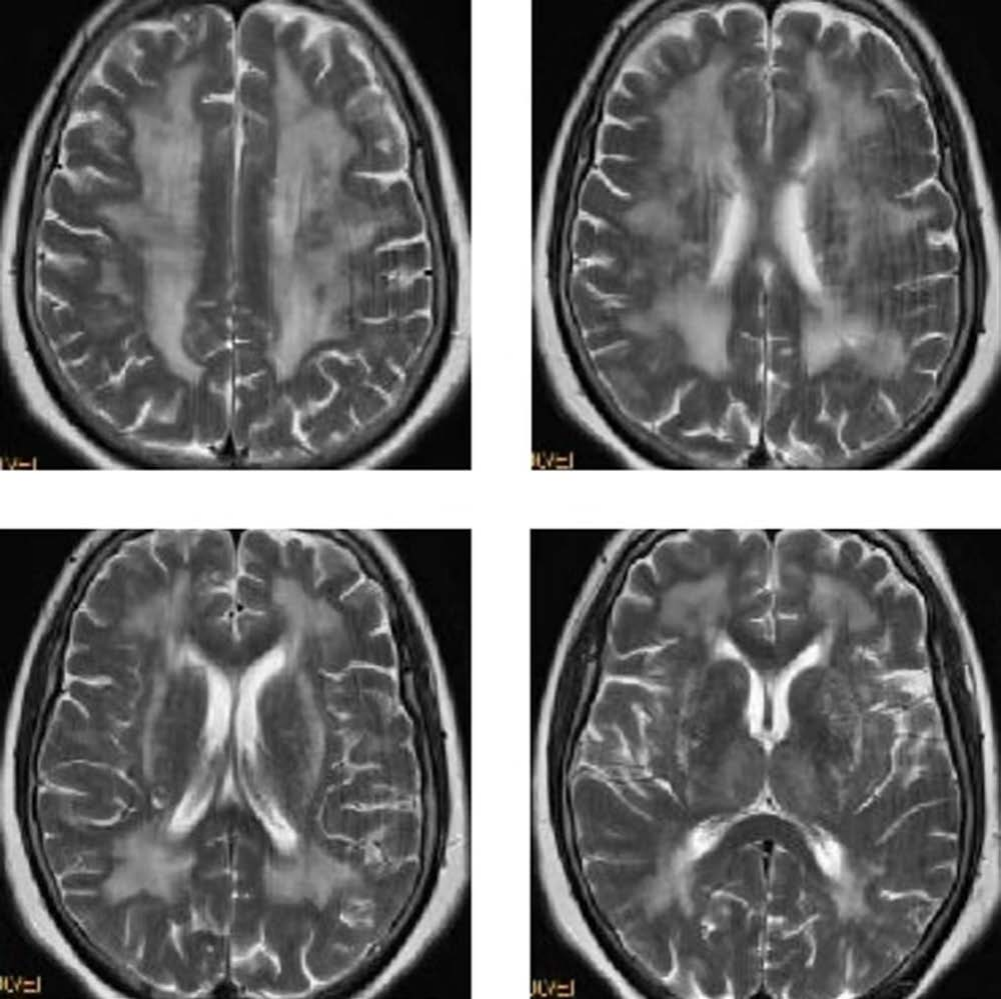
Cerebral MRI image of the patient’s mother’s brain. MRI = magnetic resonance imaging.

**Figure 3.**
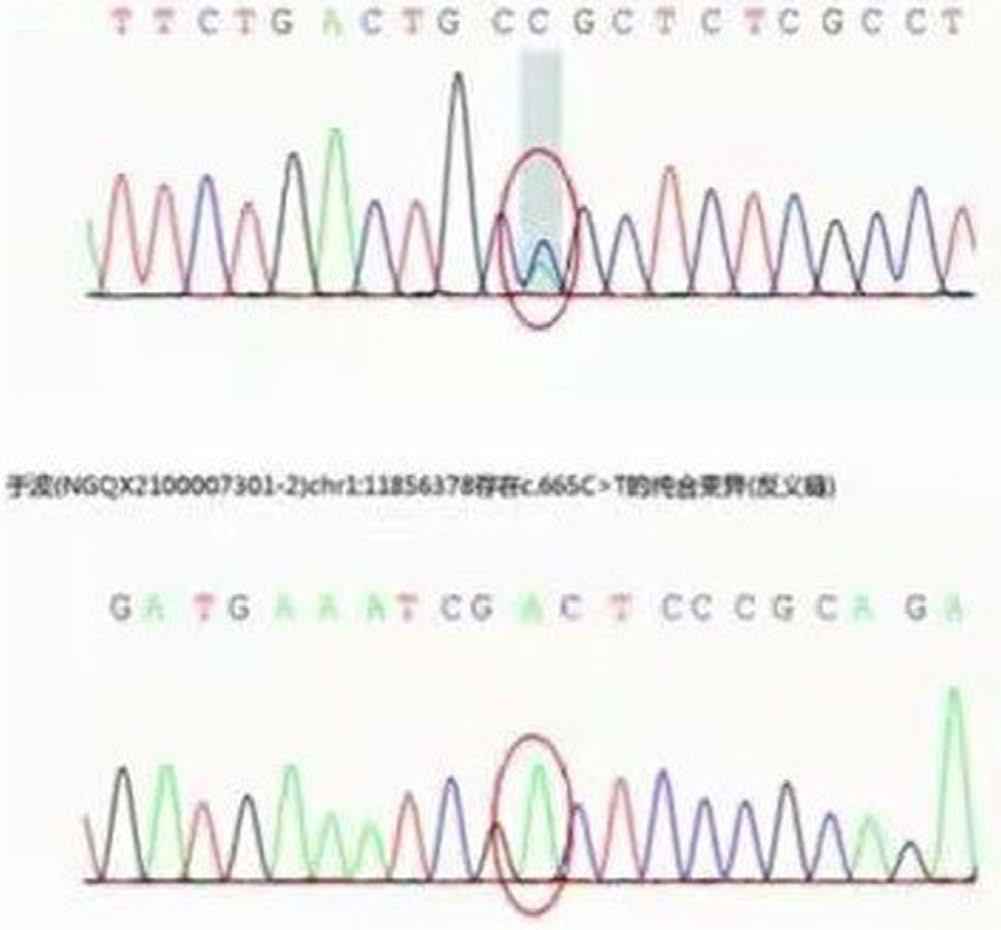
Genetic test results of the patient.

Further ask if any close relatives of the patient had undergone cerebral imaging examination. It was found that her mother had suffered from trauma months ago and had undergone cerebral MRI examination. The MRI examination results as show in Figure 2. Cerebral MRI of the patient’s mother showed bilateral symmetrical white matter lesions of the anteroposterior and posteroposterior side of the lateral ventricles and the parabody of the lateral ventricles, which were connected into slices. The patient’s mother had the same pattern of white matter lesions as the patient, but the degree of lesions is heavier than that of patients. Therefore, we speculated that the patient had a high possibility of hereditary white matter lesions. Table 1 shows the results of further genetic testing of patient. Figure 3 shows that the patient with a heterozygous mutation c.931T > G (thymine > guanine), resulting in the change of amino acid p.C311G (cysteine > glycine).

**Table 1:** Results of further genetic testing of patient.

Gene	*NOTCH3*	*MTHFR*
Variable site	c.931T > G chr19-15302340 p.C311G	c.665C > T chr1-11856378 p.A222V
Zygote	Heterozygosis 135/176 0.57	Isozygoty 0/401 1.00
Carrying rate of normal population	-	0.3085568
Transcription version gene subregion	NM_000435.2 exon6	NM_005957.4 exon5
Family validation	-	-
ACMG variant rating	Pathogenic	VUS
Disease information	1. Cerebral arteriosis with subcortical infarction and leukoencephalopathy type 1 (AD); 2. Lateral meningocele syndrome (AD); 3. Myofibromatosis in infants type 2 (AD)	1. MTHFR-deficient homocysteinuria (AR)

## 3. Discussion

The patient had a heterozygous missense mutation on exon 6 of *NOTCH3* gene, which resulted in amino acid changes p.C311G (Cysteine > Glycine) in our study. According to ACMG guidelines, this variation can be rated as pathogenic.^[9]^ However, HGMDpro database has not been reported until now. The patient did not show any typical clinical manifestations of CADASIL, and patient’s mother only showed mild cognitive dysfunction, with no obvious restriction on her daily life. However, MRI of the brain of the patient and her mother conformed to the characteristics of CADASIL. Compared with classical CADASIL, the mutation caused by this mutation site shows relatively mild CADASIL clinical symptoms.

*NOTCH3* gene mainly encodes highly conserved transmembrane receptors and participates in specific cell death during embryonic development. The gene contains 33 exons and encodes a 2321 amino acid transmembrane receptor. Its extracellular domain contains 34 EGF-like repeats, which are encoded by exons from 2 to 23.^[10]^ The study showed that CADASIL-related mutations were mainly distributed in these 34 EGF-like repeats.^[11]^ Wang et al^[12]^ report that in 33 CADASIL Chinese families, 85% of the mutations were located on exons 3 or 4, and the rest were located on exons 2, 11 or 20, in addition, some researchers reported cases of CADASIL caused by mutations on exon 6.

Gene mutation causes the increase or decrease of cysteine residues in EGFR, which makes the number of cysteine residues become singular, thus destroying the structure of disulfide bond.^[13]^ That *NOTCH3* protein, encoded by the mutated gene, forms extracellular aggregates and deposits in smooth muscle cells in the walls of small arteries, including brain, kidney, spleen, heart muscle, muscle, and cerebral arterioles.^[14]^ This phenomenon can lead to subcortical infarction and diffuse white matter hyperintensity, and white matter lesions in the temporal lobe or external capsule are characteristic for the diagnosis of CADASIL disease.^[15,16]^ Mutations not only affect the formation of disulfide bonds in the EGFR region and destroy the stability of the extracellular segment of the transmembrane protein,^[17]^ but also affect the intracellular signal transduction of *NOTCH3* protein.^[18]^ Therefore, mutations at different *NOTCH3* gene loci can lead to different clinical symptoms.

## 4. Conclusion

This case suggests that *NOTCH3* gene mutation shows different clinical symptoms due to different mutation sites, which may be related to different effects of different mutation sites on *NOTCH3* protein intracellular signal transduction. In this study, this patient had the typical imaging findings of CADASIL (structural changes) but not the typical clinical findings of CADASIL (functional findings). Therefore, further study on the changes of different signal transduction pathways caused by *NOTCH3* gene mutation will help to better understand CADASIL and seek clinical treatment methods.

## Author contributions

**Data curation:** Yuxiang Qi.

**Formal analysis:** Hairong Li.

**Methodology:** Hairong Li.

**Investigation:** Ling Yu.

**Writing – original draft:** Yuxiang Qi, Ling Yu.

**Writing – review & editing:** Yuxiang Qi, Ling Yu.
